# Two new species of *Itagonia* Reitter (Coleoptera, Tenebrionidae, Blaptini) from China

**DOI:** 10.3897/zookeys.309.5499

**Published:** 2013-06-13

**Authors:** Ai-min Shi

**Affiliations:** 1 Key Laboratory of Southwest China Wildlife Resources Conservation, Institute of Rare Animals & Plants, China West Normal University, Nanchong, Sichuan 637009, China

**Keywords:** Coleoptera, Tenebrionidae, *Itagonia*, new species, identification key, Sichuan, China

## Abstract

Two new species of *Itagonia* Reitter, 1887, *Itagonia tibialis* **sp. n.** and *Itagonia litangensis* **sp. n.** are described from Sichuan, China. A key to the known species of *Itagonia* from China is given.

## Introduction

**Figure 1.**
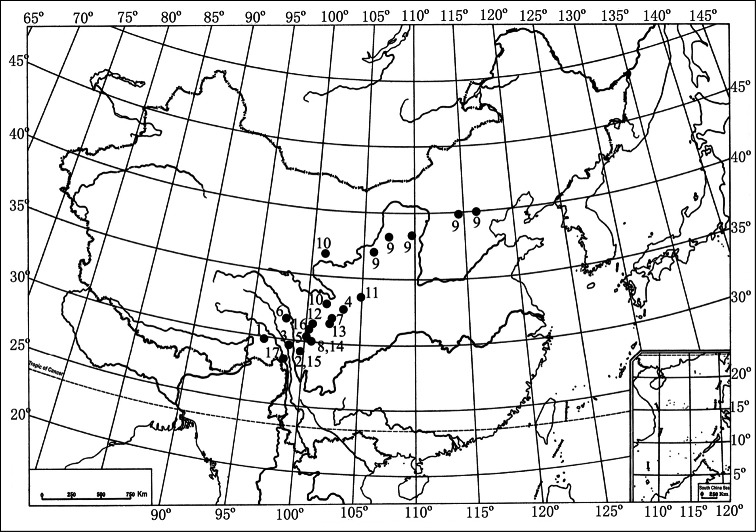
Distribution of species of the genus *Itagonia* Reitter from China: **1** *Itagonia baxoica* Liu & Ren, 2009 **2** *Itagonia bisetosa* Medvedev, 1998 **3** *Itagonia cordiformis* Shi & Ren, 2007 **4** *Itagonia elegans* Medvedev, 1998 **5** *Itagonia litangensis* sp. n. **6** *Itagonia longicornis* Shi & Ren, 2007 **7** *Itagonia medvedevi* Shi et al., 2010 **8** *Itagonia mera* Medvedev, 1998 **9** *Itagonia provostii* Fairmaire, 1888 **10** *Itagonia semenovi* Reitter, 1889 **11** *Itagonia shamaevi* Medvedev, 2004 **12** *Itagonia szetschwana* Schuster, 1923 **13** *Itagonia tibialis* sp. n. **14** *Itagonia trisetosa* Medvedev, 1998 **15** *Itagonia tuberculata* Shi et al., 2010 **16** *Itagonia xinlongensis* Shi et al., 2010 **17** *Itagonia zayica* Shi & Ren, 2007

*Itagonia* Reitter, 1887 is among the most speciose genera in the subtribe Gnaptorinina of the tribe Blaptini. It comprises 19 species and one subspecies (Reitter 1887, 1889; Fairmaire 1888; Schuster 1914, 1923; Reinig 1931; Medvedev 1998, 2004; Shi and Ren 2007a, 2007b; Egorov 2007; Liu and Ren 2009, Shi et al. 2010). Most of the species are restricted to the Hissaro-Darvaz Mountains (four species and one subspecies, representing 25% of the known taxa) and the eastern part of the Tibetan Plateau (thirteen species, representing 65% of the known taxa). *Itagonia shamaevi* Medvedev, 2004 is known from Gansu and *Itagonia provostii* (Fairmaire, 1888) from Beijing, Hebei, Neimenggu, Shaanxi and Ningxia (Fig. 1).

In this study, we follow the classification of Medvedev and Merkl (2002). Within the subtribe Gnaptorinina, the genus *Itagonia* is diagnosed by the following characters: upper edge of inner surface of profemur with tooth or obtuse-angled or arcuate prominence; upper spur of male protibiae larger than the lower spur, that of female protibiae massive, digitiform, the lower spur very small or invisible; apicale of aedeagus with outer margins arcuately or evenly narrowing to apex, or with outer margins slightly sinuate.

During the identification of tenebrionid specimens collected in Sichuan in 2012, two new species of the genus *Itagonia*, *Itagonia tibialis* sp. n. from Jinchuan and *Itagonia litangensis* sp. n. from Litang were found, which are described below.

## Material and methods

All specimens were examined and measured under a Leica M205C stereomicroscope. Drawings of the new species were prepared under the same microscope equipped with a drawing tube. The photos were taken with a Canon PowerShot SX150IS digital camera. All specimens studied are deposited in the Museum of China West Normal University (MCWNU), Nanchong, China.

## Taxonomy

### Key to the species of the genus *Itagonia* from China

**Table:** 

1	Upper edge of inner surface of profemur (figs 1–5 in Shi et al. 2010; Fig. 19) with tooth near apex	2
–	Upper edge of inner surface of profemur with arcuate or obtuse-angled prominence (figs 6–10, 14, 27, 40 in Shi et al. 2010; Fig. 33) near apex	8
2	Outer margin of epipleura visible from above along or nearly along entire length	3
–	Outer margin of epipleura visible from above at most in anterior 1/2 and/or apex	4
3	Tooth of profemur obtuse. Lateral margins of pronotum distinctly bordered before base. Apicale of aedeagus (Fig. 2) less elongate, 1.83 times as long as wide, with outer margins slightly sinuate near middle	*Itagonia szetschwana* Schuster, 1923
–	Tooth of profemur sharply acute-angled. Lateral margins of pronotum very finely, almost invisibly bordered. Apicale of aedeagus (Fig. 3) more elongate, 2.61 times as long as wide, with outer margins nearly straight	*Itagonia semenowi* Reitter, 1889
4	Only apical part of outer margin of epipleura visible from above	*Itagonia shamaevi* Medvedev, 2004
–	At least part of anterior 1/2 of outer margin of epipleura visible from above	5
5	Each elytron with traces of two longitudinal carinae, dense granules and sparse irregular prominences. Inner surface of male protibiae with arcuate prominence near base	*Itagonia tibialis* sp. n.
–	Elytra with punctures and wrinkles. Inner surface of male protibiae straight near base	6
6	Pronotum more transverse, 1.40 times as wide as long, widest behind middle. Only plantar surface of protarsomere 1 with hair brush. Apicale of aedeagus (Fig. 4) with outer margins nearly straight	*Itagonia provostii* (Fairmaire, 1888)
–	Pronotum less transverse, 1.15–1.24 times as wide as long, widest in middle. Plantar surface of protarsomeres 1 and 2 and mesotarsomere 1 with hair brushes. Apicale of aedeagus (Figs 5, 6) with outer margins sinuate	7
7	Lateral margins of pronotum converging to base with almost straight line in basal half. Prosternum in front of procoxae oblique to horizontal plane. Metatibiae straight. Apicale of aedeagus (Fig. 5) with outer margins more sinuate near middle	*Itagonia zayica* Shi & Ren, 2007
–	Lateral margins of pronotum almost parallel in basal half. Prosternum in front of procoxae almost vertical. Metatibiae weakly incurved. Apicale of aedeagus (Fig. 6) with outer margins less sinuate in apical 1/3	*Itagonia baxoica* Liu & Ren, 2009
8	Outer margin of epipleura visible from above along entire length	9
–	Outer margin of epipleura only partly visible from above	12
9	Lateral margins of pronotum distinctly reflexed. Plantar surface of pro- and mesotarsomeres without hair brush or only plantar surface of protarsomere 1 with small hair brush. Apicale of aedeagus (Fig. 7) with outer margins weakly sinuate before middle	*Itagonia elegans* Medvedev, 1998
–	Lateral margins of pronotum not reflexed. At least plantar surface of protarsomeres 1 and 2 with hair brushes. Apicale of aedeagus with outer margins weakly sinuate near middle (Fig. 8), smoothly converging from base to apex (Fig. 9) or sinuate in basal 1/3 (Fig. 10)	10
10	Antennae longer, surpassing beyond pronotal base. Upper spur of protibiae slightly longer than the lower spur	*Itagonia longicornis* Shi & Ren, 2007
–	Antennae shorter, not reaching or reaching, but not surpassing pronotal base. Upper spur of protibiae significantly longer than the lower spur	11
11	Anterior margin of pronotum more sinuate, lateral margins rectilinearly converging toward base in basal 1/2. Upper edge of inner surface of profemur with massive arcuate prominence near apex. Plantar surface of mesotarsomere 1 with apical tuft of light setae. Apicale of aedeagus (Fig. 9) with outer margins smoothly converging from base to apex	*Itagonia bisetosa* Medvedev, 1998
–	Anterior margin of pronotum less sinuate, lateral margins slightly sinuate in basal 1/4. Upper edge of inner surface of profemur with obtuse-angled prominence near apex. Plantar surface of mesotarsomere 1 with small hair brush. Apicale of aedeagus (Fig. 10) with outer margins sinuate in basal 1/3, and apical part regularly narrowing towards apex	*Itagonia xinlongensis* Shi et al., 2010
12	Plantar surface of protarsomeres 1 and 2 with hair brushes or apical tuft of pale hairs	13
–	Plantar surface of protarsomeres 1 to 3 with hair brushes	14
13	Pronotum widest before base, lateral margins less arcuately protruding. Elytral surface coarse, with irregular prominences and very sparse punctures. Plantar surface of mesotarsomere 1 with hair brush. Apicale of aedeagus (Fig. 11) less elongate, 1.46 times as long as wide, with outer margins smoothly converging from base to apex	*Itagonia tuberculata* Shi et al., 2010
–	Pronotum widest before middle, lateral margins more arcuately protruding. Elytral surface smooth, with fine punctures and irregular wrinkles. Plantar surface of mesotarsomere 1 with apical tuft of pale hairs. Apicale of aedeagus (Fig. 12) more elongate, 2.0 times as long as wide, with outer margins sinuate in basal 1/4	*Itagonia cordiformis* Shi & Ren, 2007
14	Anterior 2/3 or only apical part of outer margin of epipleura visible from above	15
–	Less than anterior 1/2 of outer margin of epipleura visible from above	16
15	Pronotal surface not flattened along outer margins. Outer margin of epipleura in dorsal view concealed by outer convexity of elytra only in apical 1/3. Apicale of aedeagus (Fig. 13) with outer margins smoothly converging from base to apex. Body significantly smaller, length 9.6 mm	*Itagonia trisetosa* Medvedev, 1998
–	Pronotal surface widely flattened along outer margins in basal half. Outer margin of epipleura visible from above only in apex. Apicale of aedeagus (Fig. 14) with outer margins slightly sinuate in apical part. Body large, length 10.8–11.7 mm	*Itagonia mera* Medvedev, 1998
16	Prosternum in front of procoxae oblique to horizontal plane. Apical part of outer margin of epipleura visible from above. Upper spur of protibiae massive, longer than protarsomere 1. Plantar surface of mesotarsomere without hair brush. Apicale of aedeagus (Fig. 15) 1.51 times as long as wide	*Itagonia medvedevi* Shi et al., 2010
–	Prosternum in front of procoxae steeply sloping. Apical part of outer margin of epipleura invisible from above. Upper spur of protibiae not massive, shorter than protarsomere 1. Plantar surface of mesotarsomeres 1 to 2 with hair brushes. Apicale of aedeagus (Fig. 36) 1.43 times as long as wide	*Itagonia litangensis* sp. n.

#### Itagonia tibialis sp. n.

urn:lsid:zoobank.org:act:0A1C342C-D3B4-44D7-B98A-D96D08FC5720

http://species-id.net/wiki/Itagonia_tibialis

Figs 16–29 , 44–45

##### Type material.

Holotype male: China, Sichuan, Jinchuan, 31°29'N, 102°05'E, 2647 m, 31 Jul. 2012, Y. C. Li and Y. P. Lai leg. (MCWNU). Paratypes: 9 males and 5 females, same data as the holotype (MCWNU).

##### Diagnosis.

This new species can be distinguished from other *Itagonia* species by the following differences: each elytron with traces of two longitudinal carinae; inner surface of male protibiae with arcuate prominence near base. *Itagonia tibialis* sp. n. belongs to the group including also *Itagonia bisetosa* Medvedev, 1998, *Itagonia tuberculata* Shi et al., 2010 and *Itagonia trisetosa* Medvedev, 1998. Representatives of this group differ from other *Itagonia* species in having apicale of aedeagus flat, smoothly tapering from base to apex, forming no sharp narrowing in apical part (Figs 9, 11, 13, 22). Occurring together with *Itagonia bisetosa*, *Itagonia tuberculata* and *Itagonia trisetosa*, the new species can be distinguished by upper edge of inner surface of profemur forming in apical part rectangular tooth. Also, the described species sharply differs from *Itagonia bisetosa* and *Itagonia trisetosa* in having less than anterior 1/2 of outer margin of epipleura visible from above, and from *Itagonia tuberculata* in having the pronotum widest before middle.

##### Etymology.

Named after the protibiae of male, inner surface of which has an arcuate prominence near base. This sharply differs from those of other species of *Itagonia*.

##### Description.

Body black, elytra dull, other parts of body weakly shining.

**Male** (Figs 16, 18–26). Anterior margin of clypeus weakly sinuate. Lateral margin of head almost without incision above antennal base. Genal margin parallel before eyes. Eyes not protruding beyond contour of head. Vertex slightly convex or flat, with moderately dense punctures. Frontoclypeal suture very shallow or invisible. Antennae (Fig. 16) reaching or nearly reaching pronotal base. Length (width) ratio of antennomeres 2 to 11 as follows: 18(17): 67(18): 25(16): 27(17): 31(18): 31(18): 23(22): 22(24): 21(25): 31(23).

Pronotum (Fig. 18) 1.15–1.24 (1.19 on average, n=10) times as wide as long, maximum width before middle, 1.63–1.77 (1.68 on average, n=10) times as wide as head. Ratio of pronotal width at anterior margin to its maximum width and width at base (n=10) 0.55: 1.00: 0.94 on average. Lateral margins of pronotum sharply arcuately converging to anterior margin in anterior 1/3, slightly narrowing to base in basal half or nearly parallel in basal 1/4, entirely bordered. Anterior margin of pronotum weakly sinuate, bordered laterally; base straight, not bordered. Anterior angles of pronotum weakly obtuse, rounded apically; posterior ones weakly obtuse or nearly rectangular. Pronotal surface between lateral margins weakly convex, with shallow median depression at disc; punctures as large as those on head, fine at disc center and larger laterally. Propleura concave in basal half, with wrinkles and very sparse granules. Prosternum in front of procoxae gently sloping; intercoxal process with shallow median depression, steeply sloping behind procoxae.

Elytra elongate-oval, 1.56–1.65 (1.60 on average, n=10) times as long as wide, maximum width in anterior 1/3, 1.35–1.44 (1.41 on average, n=10) times as wide as pronotum. Less than anterior 1/2 of outer margin of epipleura visible from above. Elytral surface between epipleura and sutural margin convex. Each elytron with traces of 2 longitudinal carinae, dense granules and sparse irregular prominences. Epipleural surface smooth, with sparse wrinkles and very sparse granules. Abdominal ventrites with punctures and short brown setae, abdominal ventrites 1 to 3 with longitudinal wrinkles, basal two abdominal ventrites with concave impression in middle.

Legs (Figs 19–21) moderately robust, length (width) ratio of pro-, meso- and metafemora 74(26): 74(19): 100(21); tibiae: 70(11): 63(13): 95(14). Upper edge of inner surface of profemur with rectangular tooth in apical part. Inner surface of protibiae with arcuate prominence near base. Upper spur of protibiae not very massive, shorter than protarsomere 1, lower spur fine and pointed. Plantar surface of protarsomeres 1 and 2 and mesotarsomere 1 with hair brushes. Metatibiae weakly incurved, regularly widening apicad. Length (width) ratio of metatarsomeres 1 to 4 as follows: 25(6.4): 12(6.0): 12(5.7): 22(6.4).

Aedeagus (Figs 22–24): length 3.83 mm, width 0.79 mm. Apicale 1.33 mm long and 0.70 mm wide, with outer margins arcuately narrowing to apex. Spiculum gastrale as in Fig. 25. Apical margin of abdominal ventrite 8 sinuate (Fig. 26).

**Female** (Figs 17, 27–29). Body wider. Antennae (Fig. 17) shorter than in male. Pronotum 1.24–1.33 (1.28 on average, n=5) times as wide as long. Elytra 1.41–1.48 (1.44 on average, n=5) times as long as wide. Less than anterior 1/3 of outer margin of epipleura visible from above. Protibiae nearly straight. Upper spur of protibiae massive and rounded apically; lower spur fine. Metatibiae straight. Plantar surface of protarsomeres and mesotarsomeres without brush. Ovipositor as in Figs 27–28. Spiculum ventrale as in Fig. 29.

##### Measurements.

Male body length 13.4–15.1 mm, width 5.4–6.3 mm; female body length 13.8–15.6 mm, width 6.7–7.2 mm.

**Figures 2–15.**
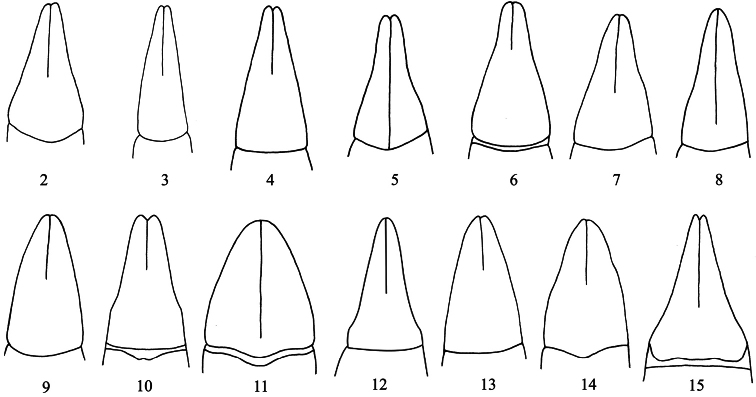
Apicale of aedeagus in dorsal view. **2** *Itagonia szetschwana* Schuster, 1923 **3** *Itagonia semenowi* Reitter, 1889 **4** *Itagonia provostii* (Fairmaire, 1888) **5** *Itagonia zayica* Shi & Ren, 2007 **6** *Itagonia baxoica* Liu & Ren, 2009 **7** *Itagonia elegans* Medvedev, 1998 **8** *Itagonia longicornis* Shi & Ren, 2007 **9** *Itagonia bisetosa* Medvedev, 1998 **10** *Itagonia xinlongensis* Shi et al., 2010 **11** *Itagonia tuberculata* Shi et al., 2010 **12** *Itagonia cordiformis* Shi & Ren, 2007 **13** *Itagonia trisetosa* Medvedev, 1998 **14** *Itagonia mera* Medvedev, 1998 **15** *Itagonia medvedevi* Shi et al., 2010. (figs 2–3 from Medvedev 2001; figs 4 and 6 from from Liu and Ren 2009; figs 7, 9 and 13–14 from Medvedev 1998)

**Figures 16–29.**
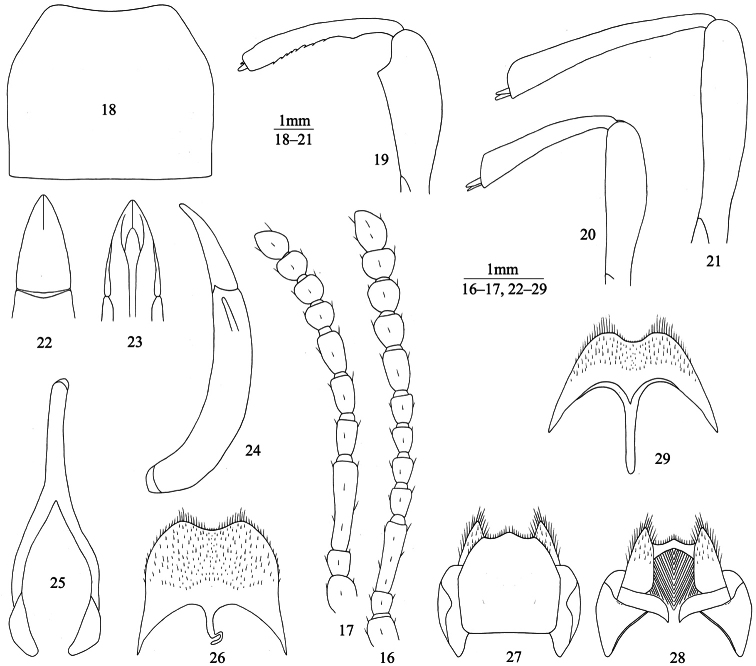
*Itagonia tibialis* sp. n. **16** antenna of male **17** antenna of female **18** pronotum of male **19** profemur and protibiae of male **20** mesofemur and mesotibiae of male **21** mtafemur and metatibiae of male **22–23** apicale of aedeagus in dorsal and ventral views **24** aedeagus in lateral view **25** spiculum gastrale **26** abdominal ventrite 8 of male **27–28** ovipositor in dorsal and ventral views **29** spiculum ventrale.

#### Itagonia litangensis sp. n.

urn:lsid:zoobank.org:act:6964C433-F497-425E-A637-4D06DD365417

http://species-id.net/wiki/Itagonia_litangensis

Figs 30–43 , 46–47

##### Type material.

Holotype male: China, Sichuan, Litang, 30°18'N, 100°16'E, 3410 m, 2 Aug. 2012, A. M. Shi leg. (MCWNU). Paratypes: 5 males and 4 females, same data as the holotype (MCWNU).

##### Diagnosis.

The new species resembles *Itagonia medvedevi* Shi et al., 2010 and *Itagonia cordiformis* Shi & Ren, 2007 by less than anterior 1/2 of outer margin of epipleura visible from above and apicale of aedeagus with outer margins sinuate, apical part rather sharply narrowing apicad (Figs 12, 15, 36). *Itagonia litangensis* sp. n. differs from *Itagonia medvedevi* and *Itagonia cordiformis* in having the prosternum in front of procoxae steeply sloping; upper spur of protibiae not massive, shorter than protarsomere 1; plantar surface of mesotarsomeres 1 to 2 with hair brushes; apical margin of abdominal sternite 8 weakly sinuate. Also, the new species can be distinguished from *Itagonia medvedevi* by apical part of outer margin of epipleura invisible from above, and from *Itagonia cordiformis* by lateral margins of pronotum weakly arcuately protruding.

##### Etymology.

Named after the type locality, Litang.

##### Description.

Body black, weakly shining.

**Figures 30–43.**
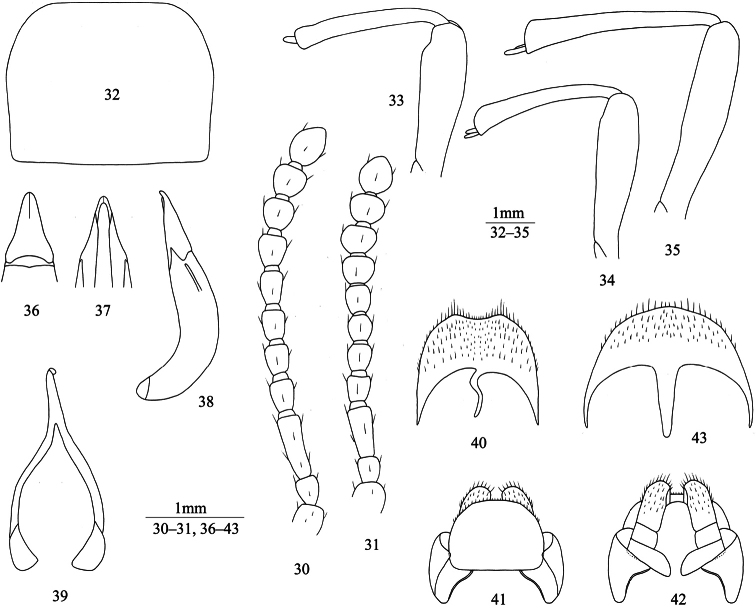
*Itagonia litangensis* sp. n. **30** antenna of male **31** antenna of female **32** pronotum of male **33** profemur and protibiae of male **34** mesofemur and mesotibiae of male **35** mtafemur and metatibiae of male **36–37** apicale of aedeagus in dorsal and ventral views **38** aedeagus in lateral view **39** spiculum gastrale **40** abdominal ventrite 8 of male **41–42** ovipositor in dorsal and ventral views **43** spiculum ventrale.

**Male** (Figs 30, 32–40). Anterior margin of clypeus nearly straight. Lateral margin of head with obtuse-angled shallow incision above antennal base. Genal margin arcuately converging to clypeal base. Eyes not protruding beyond contour of head. Vertex slightly convex, with moderately dense punctures. Frontoclypeal suture shallow. Antennae (Fig. 30) reaching posterior 1/4 of pronotum. Length (width) ratio of antennomeres 2 to 11 as follows: 16(13): 33(14): 18(14): 18(14): 18(14): 20(14): 18(17): 18(19): 18(20): 25(19).

Pronotum (Fig. 32) transverse, 1.25–1.35 (1.28 on average, n=6) times as wide as long, maximum width before middle, 1.77–1.86 (1.84 on average, n=6) times as wide as head. Ratio of pronotal width at anterior margin to its maximum width and width at base (n=6) 0.67: 1.00: 0.91 on average. Lateral margins of pronotum more sharply arcuately narrowing to anterior margin than to base, entirely bordered. Anterior margin of pronotum nearly straight; base straight, both bordered laterally. Anterior angles of pronotum obtuse, rounded apically; posterior ones weakly obtuse. Pronotal surface between lateral margins convex, with short median depression at disc; punctures as large as those on head, fine at disc center and larger laterally. Propleura slightly concave, with wrinkles and very sparse granules. Prosternum in front of procoxae steeply sloping; intercoxal process with shallow median depression, steeply sloping behind procoxae.

Elytra elongate-oval, 1.35–1.42 (1.39 on average, n=6) times as long as wide, maximum width before middle, 1.21–1.31 (1.25 on average, n=6) times as wide as pronotum. Less than anterior 1/2 of outer margin of epipleura visible from above. Elytral surface between outer margin of epipleura and sutural margin convex, sparsely covered with irregular fine wrinkles and fine punctures. Epipleural surface with sparse wrinkles. Abdominal ventrites with punctures and brown setae, abdominal ventrites 1 to 3 with longitudinal wrinkles.

Legs (Figs 33–35) robust, length (width) ratio of pro-, meso- and metafemora 78(22): 82(20): 100(21); tibiae: 74(11): 71(12): 94(16). Upper spur of protibiae not massive, shorter than protarsomere 1, lower spur fine and pointed. Plantar surface of protarsomeres 1 to 3 and mesotarsomeres 1 to 2 with hair brushes. Metatibiae weakly incurved. Length (width) ratio of metatarsomeres 1 to 4 as follows: 24(8.0): 13(7.5): 12(6.7): 25(6.7).

Aedeagus (Figs 36–38): length 2.24 mm, width 0.71 mm. Apicale 0.69 mm long and 0.48 mm wide, with outer margins slightly sinuate in basal 1/3. Spiculum gastrale as in Fig. 39. Apical margin of abdominal ventrite 8 weakly sinuate (Fig. 40).

**Female** (Figs 31, 41–43). Body wider. Antennae (Fig. 31) shorter than in male. Pronotum 1.27–1.35 (1.32 on average, n=4) times as wide as long. Elytra 1.27–1.35 (1.31 on average, n=4) times as long as wide. Less than anterior 1/3 of outer margin of epipleura visible from above. Upper spur of protibiae massive and rounded apically; lower spur missing. Plantar surface of protarsomeres and mesotarsomeres without brush. Ovipositor as in Figs 41–42. Spiculum ventrale as in Fig. 43.

##### Measurements.

Male body length 11.0–12.2 mm, width 5.2–5.8 mm; female body length 11.4–12.1 mm, width 5.6–6.1 mm.

**Figures 44–47.**
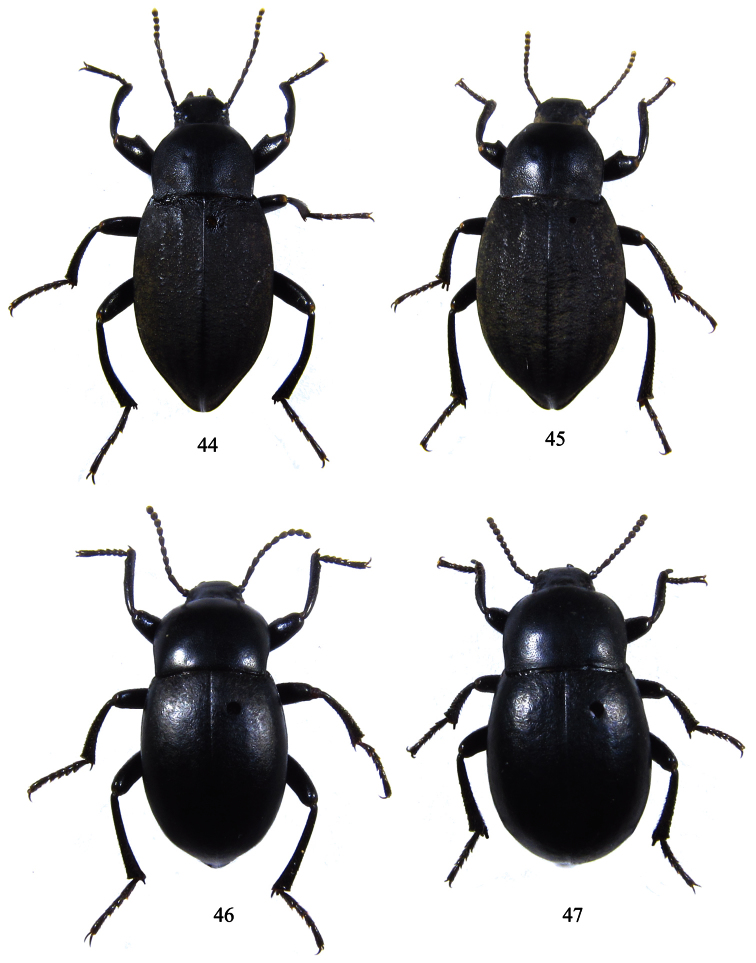
**44–45** *Itagonia tibialis* sp. n. **44** male, length 14.2 mm **45** female, length 14.9 mm **46–47** *Itagonia litangensis* sp. n. **46** male, length 11.5 mm **47** female, length 11.8 mm.

## Supplementary Material

XML Treatment for Itagonia tibialis

XML Treatment for Itagonia litangensis
